# A science-based agenda for health-protective chemical assessments and decisions: overview and consensus statement

**DOI:** 10.1186/s12940-022-00930-3

**Published:** 2023-01-12

**Authors:** Tracey J. Woodruff, Swati D. G. Rayasam, Daniel A. Axelrad, Patricia D. Koman, Nicholas Chartres, Deborah H. Bennett, Linda S. Birnbaum, Phil Brown, Courtney C. Carignan, Courtney Cooper, Carl F. Cranor, Miriam L. Diamond, Shari Franjevic, Eve C. Gartner, Dale Hattis, Russ Hauser, Wendy Heiger-Bernays, Rashmi Joglekar, Juleen Lam, Jonathan I. Levy, Patrick M. MacRoy, Maricel V. Maffini, Emily C. Marquez, Rachel Morello-Frosch, Keeve E. Nachman, Greylin H. Nielsen, Catherine Oksas, Dimitri Panagopoulos Abrahamsson, Heather B. Patisaul, Sharyle Patton, Joshua F. Robinson, Kathryn M. Rodgers, Mark S. Rossi, Ruthann A. Rudel, Jennifer B. Sass, Sheela Sathyanarayana, Ted Schettler, Rachel M. Shaffer, Bhavna Shamasunder, Peggy M. Shepard, Kristin Shrader-Frechette, Gina M. Solomon, Wilma A. Subra, Laura N. Vandenberg, Julia R. Varshavsky, Roberta F. White, Ken Zarker, Lauren Zeise

**Affiliations:** 1 Program On Reproductive Health and the Environment, Department of Obstetrics, Gynecology and Reproductive Sciences, University of California, San Francisco, 490 Illinois Street, Floor 10, Box 0132, San Francisco, CA 94143 USA; 2 Independent Consultant, Washington, DC USA; 3 Department of Environmental Health Sciences, School of Public Health, University of Michigan, Ann Arbor, MI USA; 4 Department of Public Health Sciences, University of California, Davis, Davis, CA USA; 5 National Institutes of Environmental Health Sciences and National Toxicology Program, Research Triangle Park, NC USA; 6 Duke University, Durham, NC USA; 7 Social Science Environmental Health Research Institute, Northeastern University, Boston, MA USA; 8 Department of Food Science and Human Nutrition, Department of Pharmacology and Toxicology, Michigan State University, East Lansing, MI USA; 9 Department of Philosophy, University of California, Riverside, Riverside, CA USA; 10 Environmental Toxicology Graduate Program, College of Natural and Agricultural Sciences, University of California, Riverside, Riverside, CA USA; 11 Department of Earth Sciences, University of Toronto, Toronto, ON Canada; 12 School of the Environment, University of Toronto, Toronto, ON Canada; 13 Clean Production Action, Somerville, MA USA; 14 Earthjustice, New York, NY USA; 15 The George Perkins Marsh Institute, Clark University, Worcester, MA USA; 16 Department of Environmental Health, T.H. Chan School of Public Health, Harvard University, Boston, MA USA; 17 Department of Environmental Health, School of Public Health, Boston University, Boston, MA USA; 18 Department of Public Health, California State University, East Bay, Hayward, CA USA; 19 Defend Our Health, Portland, ME USA; 20 Pesticide Action Network, Berkeley, CA USA; 21 School of Public Health, University of California, Berkeley, Berkeley, CA USA; 22 Department of Environmental Science, Policy and Management, University of California, Berkeley, Berkeley, CA USA; 23 Department of Environmental Health and Engineering, Bloomberg School of Public Health, Johns Hopkins University, Baltimore, MD USA; 24 Johns Hopkins Risk Sciences and Public Policy Institute, Bloomberg School of Public Health, Johns Hopkins University, Baltimore, MD USA; 25 School of Medicine, University of California, San Francisco, CA USA; 26 Department of Biological Sciences, Center for Human Health and the Environment, North Carolina State University, Raleigh, NC USA; 27 Commonweal, Bolinas, CA USA; 28 Center for Reproductive Sciences, Department of Obstetrics, Gynecology & Reproductive Sciences, University of California, San Francisco, San Francisco, CA USA; 29 Silent Spring Institute, Newton, MA USA; 30 Natural Resources Defense Council, Washington, DC USA; 31 Department of Pediatrics, University of Washington, Seattle, WA USA; 32 Department of Child Health, Behavior, and Development, Seattle Children’s Research Institute, Seattle, WA USA; 33 Science and Environmental Health Network, Ames, IA USA; 34 Department of Environmental and Occupational Health Sciences, University of Washington School of Public Health, Seattle, USA; 35 Department of Urban & Environmental Policy and Public Health, Occidental College, Los Angeles, CA USA; 36 WE ACT for Environmental Justice, New York, NY USA; 37 Department of Biological Sciences, University of Notre Dame, Notre Dame, IN USA; 38 Department of Philosophy, University of Notre Dame, Notre Dame, IN USA; 39 Public Health Institute, Oakland, CA USA; 40 Louisiana Environmental Action Network, Baton Rouge, LA USA; 41 Department of Environmental Health Sciences, School of Public Health & Health Sciences, University of Massachusetts, Amherst, Amherst, MA USA; 42 Department of Health Sciences, Northeastern University, Boston, MA USA; 43 Department of Civil and Environmental Engineering, Northeastern University, Boston, MA USA; 44 Washington State Department of Ecology, Olympia, WA USA; 45 Office of Environmental Health Hazard Assessment, California Environmental Protection Agency, Oakland, CA USA

**Keywords:** Chemicals, Conflicts of Interest, Environmental Health, Environmental Justice, EPA, Hazard Identification, Health Equity, Risk Assessment, TSCA

## Abstract

The manufacture and production of industrial chemicals continues to increase, with hundreds of thousands of chemicals and chemical mixtures used worldwide, leading to widespread population exposures and resultant health impacts. Low-wealth communities and communities of color often bear disproportionate burdens of exposure and impact; all compounded by regulatory delays to the detriment of public health. Multiple authoritative bodies and scientific consensus groups have called for actions to prevent harmful exposures via improved policy approaches. We worked across multiple disciplines to develop consensus recommendations for health-protective, scientific approaches to reduce harmful chemical exposures, which can be applied to current US policies governing industrial chemicals and environmental pollutants. This consensus identifies five principles and scientific recommendations for improving how agencies like the US Environmental Protection Agency (EPA) approach and conduct hazard and risk assessment and risk management analyses: (1) the financial burden of data generation for any given chemical on (or to be introduced to) the market should be on the chemical producers that benefit from their production and use; (2) lack of data does not equate to lack of hazard, exposure, or risk; (3) populations at greater risk, including those that are more susceptible or more highly exposed, must be better identified and protected to account for their real-world risks; (4) hazard and risk assessments should not assume existence of a “safe” or “no-risk” level of chemical exposure in the diverse general population; and (5) hazard and risk assessments must evaluate and account for financial conflicts of interest in the body of evidence. While many of these recommendations focus specifically on the EPA, they are general principles for environmental health that could be adopted by any agency or entity engaged in exposure, hazard, and risk assessment. We also detail recommendations for four priority areas in companion papers (exposure assessment methods, human variability assessment, methods for quantifying non-cancer health outcomes, and a framework for defining chemical classes). These recommendations constitute key steps for improved evidence-based environmental health decision-making and public health protection.

## Introduction

Chemical pollution is a worldwide crisis that threatens global ecosystems, food security, and human health and reproduction [1–4]. However, the manufacture and production of industrial chemicals has continued to increase annually, with over 350,000 chemicals and chemical mixtures registered for production and use worldwide, and thousands of high production volume chemicals (1 million pounds/year) in widespread use in the United States (US) [1, 5–10]. Data demonstrate extensive population exposure to environmental pollutants, with low-wealth communities and communities of color often bearing disproportionate burdens of exposure [11–13]. Exposures to chemicals of concern increases the risk of a range of adverse health effects and chronic diseases such as cancer, neurodevelopmental dysfunction, asthma, diabetes and other metabolic diseases, immune system dysregulation, high cholesterol, and reproductive disorders, all of which have been increasing in prevalence over the last several decades [14–17]. Global concerns regarding chemical risks have continued to grow. The World Health Organization (WHO) estimated that chemical exposures resulted in two million lives and fifty-three million disability-adjusted-life-years lost in 2019 based on methods that do not fully capture complete risks [18]. Additionally, *The Lancet* Commission on Pollution and Health reported that chemicals are responsible for at least 1.8 million deaths each year globally, the majority of which occur in the Global South [3]. Due to the advance in scientific methods, multiple authoritative bodies and scientific consensus groups have recognized the impacts of environmental pollution on health and have called for actions to prevent harmful exposures including improved policy approaches to more efficiently and effectively address the safety of widespread industrial chemical use [17, 19–23]. Grassroots organizers from affected communities have also played a significant role in collaborating with public health researchers and environmental health scientists to elevate these problems and influence policy change [24, 25].

Varying policy frameworks are currently applied to address chemicals in commerce and industrial pollution [26]. Defining features of different policy approaches include whether industry or government has the responsibility to generate and evaluate data on chemical hazard and risk; to what extent these data must be disclosed to the public; whether decision-making is based on hazard or risk; and the extent to which consideration of costs is required, allowed or excluded in selecting risk management measures. In the European Union (EU), under the Registration, Evaluation, Authorisation and Restriction of Chemicals (REACH), the obligation is on chemical manufacturers and importers to generate data on potential chemical exposure and toxicity, to use this information to develop and apply appropriate risk management measures, to communicate these measures to users of chemicals and, finally, to submit this information to the European Chemicals Agency (ECHA), the EU body that will ultimately make the regulatory decision [27]. The EU’s adoption of REACH in 2007 placed the responsibility for identifying and addressing harms associated with chemical exposure on industry [28].

In the United States, multiple laws govern environmental chemicals. This includes major laws at the federal level administered by the US Environmental Protection Agency (EPA) (separate laws addressing toxic substances in commerce, pesticides, drinking water contaminants and hazardous air pollutants), or by other agencies including the Occupational Safety and Health Administration (OSHA) (occupational exposure to chemicals), Food and Drug Administration (FDA) (chemicals in cosmetics, pharmaceuticals, medical devices, and food additives), and Consumer Product Safety Commission (CPSC) (chemicals in consumer products) [29]. Under most of these US laws, unlike in the EU, the onus is placed on the government to identify and request data from the industry and to evaluate potential chemical toxicity. One exception is the Federal Insecticide, Fungicide, and Rodenticide Act (FIFRA), which requires pesticide manufacturers to produce extensive scientific data, including toxicity testing; however, EPA’s frequent waivers of toxicity testing under FIFRA raise concerns and the Agency’s reliance on industry-supported studies has been critiqued by independent scientists [30–32]. Another characteristic of chemical regulation in the US is that some state and local governments have passed legislation limiting uses of particular chemicals or chemical classes resulting in a patch-work of regulatory requirements [31].

All US laws concerning risks of chemicals require some consideration of scientific information related to exposures and health hazards, with differing levels of specificity on how these should be evaluated. For example, the Food Quality Protection Act, which regulates uses of pesticides in agricultural processes on food crops, requires that EPA’s risk assessments consider children’s particular susceptibility to the harmful effects of pesticide exposures and address both the risk of aggregate exposure to a single pesticide from multiple food sources and the cumulative risk of exposure to multiple pesticides which share a common mechanism of toxicity [33]. The Toxic Substances Control Act (TSCA) is the primary authority that regulates non-pesticide chemicals, and as amended in 2016 (amended TSCA) requires EPA to determine whether chemicals already on the market pose an “unreasonable risk” by conducting evaluations of chemical exposures, hazards and risks [34]. For chemicals to be introduced into the market, manufacturers must provide the information they have on the chemical, and while there is no minimum data set required, EPA has the authority to request additional data  [34]. Further, amended TSCA requires that EPA account for risks to “potentially exposed or susceptible subpopulations (PESS)” such as children, pregnant women, and workers, and use the “best available” science [34]. However, EPA has not applied a consistent or complete approach to identifying PESS thus far and does not currently incorporate factors or utilize methodologies to approximate the combined risk resulting from exposure to multiple chemicals, and non-chemical stressors such as food insecurity, pre-existing disease, poverty, or racism, into its risk evaluations – even though doing so would reflect the “best available” scientific approach to risk assessment in many cases [35–40]. While state or local level laws can be more protective of human health, they cover only a portion of the US population and can be preempted by federal action [34].

Other federal statutes in the US, such as the Clean Air Act (CAA) and the Safe Drinking Water Act (SDWA), which regulate specific environmental media (air and drinking water respectively), also require consideration of risks of exposure to pollutants. For example, the CAA requires EPA to perform health risk assessments for regulated industrial sources of listed hazardous air pollutants (of which there are 188, a small subset of dangerous air pollutants) to prevent all unacceptable health risks including “lifetime excess cancer risks” to the individual most exposed, and to assure “an adequate margin of safety” to protect public health [41].

Despite advances in the science, the general framework in the US for hazard and risk assessment has stayed largely the same since the 1970s. Some methodological improvements have been incorporated, such as physiologically-based toxicokinetic modeling, benchmark dose modeling, and limited application of cumulative risk assessment approaches, all which focus on refinements within the existing framework but may also present their own drawbacks [42–45]. The implementation of statutes regulating chemicals and pollutants across the US federal government relies on an overall framework for using scientific evidence in hazard and risk assessment that determines the extent to which policies are protective of public health [46]. This framework has several key features originating with the development of chemical risk assessment in the 1970s and early 1980s and includes dividing health effects into cancer and noncancer outcomes, with cancer generally treated as a non-threshold physiological process (any exposure may be associated with some level of risk) and noncancer outcomes assumed to have a threshold (below which exposure is assumed to be “safe” or “no risk” for the entire exposed population), and also includes conducting assessments for one pollutant or chemical at a time [47]. This is further discussed and demonstrated in Principle 4 below and in the companion paper by Nielsen et al. in this issue.

Multiple authoritative review bodies, including the US National Academies of Sciences, Engineering, and Medicine (NASEM), the European Food Safety Authority (EFSA), and the WHO have called for improved risk assessment approaches to better account for population variability, estimation of non-cancer risk at environmentally-relevant exposure levels, and risks due to cumulative exposures to multiple chemical and non-chemical stressors [47–50]. Inadequate incorporation of current scientific understanding and principles in exposure, hazard, and risk assessment can lead to underestimation of risk and subsequent adverse consequences for public health. For example, lack of quantification of noncancer health effects results in their exclusion when agencies attempt to account for the benefits of reducing exposure to environmental chemicals, which in turn leads to inadequate public policies to protect health [51]. Regulatory agencies urgently need to improve the use of science in decision-making processes and ensure that populations are not exposed to harmful levels of chemicals, classes of chemicals, or chemical mixtures [15, 52, 53].

### A path forward to address chemical pollution

Given the impact of environmental chemical exposures on public health and the need to integrate contemporary science into decision-making in the US, we worked across multiple disciplines, including toxicology, occupational health, exposure science, epidemiology, community health, risk assessment, law, sociology, and philosophy to develop recommendations for health-protective approaches to reduce harmful chemical exposures and improving scientific methods to identify chemical harms and assess their risks. Our recommendations incorporate contemporary science, which can be applied to current policies governing industrial chemicals and environmental pollutants, specifically regarding improvements in how EPA conducts exposure, hazard, and risk assessment and risk management analyses.

Accordingly, the companion papers we contributed to this issue of *Environmental Health* span four topics from our collaborative process that we prioritized as critical to improving regulatory science policy in the US and the public’s health:

- Addressing systemic problems in current chemical exposure assessments and recommendations for improvement to better protect public health;
- Detailing the current practice to address human variability/vulnerability and recommendations for advancing how this is accounted for in chemical risk assessment;
- Applying risk assessment methods for non-cancer health outcomes that can be used to estimate the likelihood of adverse effects in the exposed population; and
- Reviewing how chemical classes are defined and used nationally and internationally and recommending a science-based framework for classes to be used in US policy and decision-making.

These manuscripts are the culmination of a multi-year process involving meetings and workgroups attended by over 40 leaders (all coauthors in this series) from academic institutions, non-governmental organizations, community groups, and government agencies. To our knowledge, these manuscripts represent the most comprehensive assessments of their type, and the most diverse breadth of expertise across environmental health, social science, and public policy disciplines.

While developing the above manuscripts, we identified five overarching principles and scientific recommendations that apply to the use of science across all areas of exposure, hazard, and risk assessment that we detail in this manuscript and weave into each of our companion papers. These are: (1) the financial burden of data generation for any given chemical on (or to be introduced to) the market should be on the chemical producers that benefit from their production and use; (2) lack of data does not equate to lack of hazard, exposure, or risk; (3) populations at greater risk, including those that are more susceptible or more highly exposed, must be better identified and protected to account for their real-world risks; (4) hazard and risk assessments should not assume existence of a “safe” or “no-risk” level of chemical exposure in the diverse general population; and (5) hazard and risk assessments must evaluate and account for financial conflicts of interest in the body of evidence.

We conclude that these changes are critical to correct existing deficiencies in exposure, hazard, and risk assessments that lead to insufficient information to identify risks, underestimates of risk, inequitable distributions of risk, and unconsented transfer of risks from manufacturers to the public. A shift in the basic framework of how science is used in decisions requires a policy change to the status quo and this series of papers can serve as a scientific statement from experts to aid in the engagement of the public health community, community groups, government regulators and policymakers, and others in their efforts to bring about change. Our five consensus principles and recommendations are detailed in the following sections and summarized in Table 1. These principles and recommendations can be applied across a wide array of community, policy, and regulatory settings at local, state, national, and international levels to incorporate the best available science and provide a stronger foundation for decision-making about exposures and health effects related to industrial chemical use and pollution.

**Table 1:** Overview of Consensus Principles and Recommendations for Health-Protective Chemical Policy

Principle	Scientific Recommendations	Policy Recommendations
The financial burden of data generation for a chemical on (or to be introduced to) the market should be on the chemical producers benefiting from their production and use	• Generate health effects data for chemicals on the market and new chemicals prior to entry on the market that allow full health assessment of workplace, consumer, fenceline, and general population exposures • Require manufacturers to provide physical and chemical properties data and chemical standards	• Ensure financial burden of data generation for chemicals (already on market or prior to entry) is borne by the manufacturer and under the supervision of the regulator
Lack of data does not equate to lack of hazard, exposure, or risk	• Develop a set of properties to assess completeness of the database for a given chemical – such as physical and chemical properties, exposure parameters for current or past uses, and health endpoints (including for highly susceptible and exposed subpopulations) and identify gaps in the existing data • Ensure adequate and up-to-date monitoring, modeling, and toxicity data • Evaluations of hazard data based on newer methods such as computational and in vitro approaches should be used primarily to identify early signals of harm, and hazard concerns should only be downgraded if there is robust evidence in multiple systems to ensure that hazard classifications are not weakened based on speculative or limited data, but rather to provide “actionable evidence” to support regulatory restrictions	• EPA should make greater use of its authority under TSCA Sects. 4, 8, and 10 to obtain data on chemicals in commerce • EPA should re-evaluate industry’s confidential business information (CBI) claims to ensure that critical hazard data are not shielded from public view • Incentivize data generation using approaches such as publishing provisional toxicity values for chemicals that apply multiple default adjustment factors to fully account for missing data • Action should be taken to mitigate exposures and evaluate the potential harmful impacts of chemicals with evidence of harm over their full life cycle, even when there is not a full hazard and risk assessment or when evidence is limited
Populations at greater risk, including those that are more susceptible or more highly exposed, must be better identified and protected to account for their real-world risks	• Explicitly identify factors that qualify groups as susceptible subpopulations • Integrate ground-truthed knowledge and community-generated data on uses, exposures, and hazards from community experts into scientific evaluations • Rely on best available methods to account for factors that increase susceptibility when identifying hazards, estimating exposures, and quantitatively estimating dose–response relationships	• EPA should use an expanded definition of “potentially exposed or susceptible subpopulations” to ensure health risks are appropriately considered and quantified • Consult with community experts early and often and incorporate their expert knowledge in hazard and risk assessments • Account for aggregate exposure and cumulative risk in all risk assessments
Hazard and risk assessments should not assume existence of a “safe” or “no-risk” level of chemical exposure in the diverse general population	• Use risk assessment methods that quantify health risks at low levels of exposures to chemicals, unless there are sufficient data to demonstrate a threshold level below which there is no risk for the entire exposed population	• Apply methods to quantify risks of non-cancer effects and replace the reference dose (RfD) and reference concentration (RfC) with a risk-specific dose
Hazard and risk assessments must evaluate and account for financial conflicts of interest (COI) in the body of evidence	• Evaluations of study quality in hazard and risk assessments should account for the potential influence on study outcomes of study authors or study sponsors with a financial conflict of interest	• Government agencies should identify and implement policies to reduce financial conflict of interest in the scientific evidence base for decision making

#### 1. The financial burden of data generation for any given chemical on (or to be introduced to) the market should be on the chemical producers that benefit from their production and use

Under most U.S. laws concerning chemical risks, the burden of data generation and proof is on federal agencies such as EPA to assess the impact of chemicals currently on or to be introduced to the market. However, this approach presents both a financial and health burden to the public [54, 55]. Chemical manufacturers have a financial stake in the production of existing chemicals and have an incentive to delay data generation to maintain or grow their market [54, 56]. Early warning signals of harm are often ignored or downplayed by those with financial stakes in the outcome of any prevention activities, leading to delays in action to the detriment of public health; as demonstrated by numerous well-characterized toxicants including tobacco smoke, lead, and air pollutants [57–59]. For example, in 1989 EPA issued a final rule under the original TSCA to ban the use of most products containing asbestos, a naturally-occurring fiber classified as carcinogenic to humans by IARC in 1977 and 1987, and widely-used in commerce despite known health impacts in asbestos factory workers as early as 1898 [60–62]. However, asbestos industry interests sued EPA over the rule, and in 1991 a court ruled that EPA’s extensive analyses of risks, costs, and benefits were not sufficient to support a ban, thus nullifying the regulation. Now 45 years since it was declared a carcinogen and after decades of exposure and subsequent health effects and deaths, chrysotile asbestos, the most common of the six mineral fibers comprising asbestos, has been proposed to be banned under amended TSCA [63–65].

The expense of data generation should be on the chemical producers that financially benefit from their production and use. For chemicals to be introduced to the market, similar approaches used in the EU under REACH should be taken, with robust chemical safety data and a chemical standard (Principle 2) submitted to a public agency for evaluation *prior* to approving a chemical to enter commerce [27, 66]. This is a key element of *preventing* introduction of chemicals that may result in harmful exposures over their full life cycle, and would help ameliorate ongoing and long term evaluations of the science *after* widespread exposure has already occurred and industry is locked into continued production [60, 67]. Similarly, for chemicals already on the market, industry should be required to provide detailed physical and chemical properties, a chemical standard, and any known uses and hazards. As outlined in Principle 2, EPA or any agency should have a list of the sufficient and comprehensive data/information that is needed for chemical evaluation. Such a list should be informed and developed by a panel of scientific experts/independent parties without any financial conflicts of interest (Principle 5) and EPA or any agency should contract independent researchers, under the supervision of the agency and financed by industry through the agency, to generate the necessary data in a timely fashion. Ultimately the decision regarding the likelihood or potential for risk or hazard posed by a given chemical, whether it is currently on the market or to be introduced, or whether a safety standard is met, should lie with the governmental agency.

#### 2. Lack of data does not equate to lack of hazard, exposure, or risk

The legacy of limited or no data has hampered the ability of US agencies to evaluate potential health hazards and risk of chemical exposures, creating a situation where most chemicals on the market do not have sufficient data to identify their harms. Additionally, even when there are early indicators of harm, which is the case for a number of chemicals highly produced, this information is not used to prevent harmful exposures. For example, polychlorinated biphenyls (PCBs) were first synthesized in 1881 and, despite evidence of adverse health effects in exposed workers in the 1930s, were widely used in commerce until the late 1970s. PCBs were only banned from new production, but not current use, in the US after demonstrated widespread distribution in the environment and severe health effects from a human poisoning event involving contaminated rice oil [60]. This and many other examples—like pervasive PFAS contamination of drinking water—illustrate that widespread chemical use without prior hazard assessment can threaten human and environmental health [68]. Repeated documentation with new methods show the presence of chemicals and exposures at levels associated with adverse outcomes, illustrating that the absence of data does not equate to absence of hazard, exposure, or risk; and that in most cases further study leads to identification of additional risks, often at lower levels of exposure [60, 67, 69, 70].

Evidence-based decision making in the US federal government regarding chemical risks is hampered by a frequent lack of fundamental descriptive data about the chemicals themselves. For example, there is not a uniform naming convention for chemicals, not every chemical has a Chemical Abstracts Service (CAS) number, some chemicals have multiple CAS numbers, and there is a lack of chemical standards (materials containing a known concentration of a chemical for use in analysis that would provide regulators with necessary scientific information) [5, 71, 72]. Timely generation of data regarding physical and chemical properties, health, exposure, manufacture, and use (or potential use) throughout the supply chain for chemicals is a necessary first component to identify hazards and effectively prevent human health risks posed by chemicals in commerce and to ensure that new chemicals are sufficiently evaluated (and their risks mitigated) prior to entry on the market. Without adequate and up-to-date monitoring, modeling, and toxicity data, critical exposures, hazards, and health effects will remain unknown to the public and unaddressed by the private sector, researchers, and government. Failure to generate data in a timely way results in regulatory agency decisions (e.g., on permit limits or risk evaluations) that are based on inadequate data that may understate risk.

To correct this gap, EPA should require data sufficient to characterize health hazards, predict potential exposures, and characterize cumulative risk, as well as chemical standards, in order to prevent or mitigate harmful health effects of chemicals and identify them in the environment. Authority for obtaining data on chemicals in commerce exists, for example, under Sects. 4, 8, and 10 of amended TSCA [34]. A potential approach could include developing a set of properties to assess completeness of the database—such as physical and chemical properties, exposure parameters (e.g., current and past uses, use locations, workplace air monitoring, release data, etc.), and health endpoints, including for susceptible and vulnerable subpopulations that have been identified as important to fully characterize the health hazards of chemicals essential for decision-making. This data set would allow EPA to evaluate the potential for a range of health effects (e.g., neurodevelopmental, reproductive, developmental, cancer) and assays which are sufficiently robust to capture increased risk of health effects in humans (e.g., dose selection, duration of exposure, critical windows of exposure, and accounting for cumulative exposures). These would include health effects due to exposures that occur during sensitive life stages (e.g., preconception, during fetal and child development, and aging), exposure data for different settings (e.g., workplace and built environment) and representing variability across populations with different risk factors. EPA could use this list to assess the availability of data and provide a public summary characterizing the “completeness of the database” for each chemical. Finally, EPA and other federal agencies can use this list to outline data gaps, identify those most critical to decision making, and request necessary data for each chemical currently on or proposed to enter the market, which would facilitate timely data generation consistent with other programs in Canada and Europe [27, 73].

There are opportunities to use newer computational approaches and more advanced biological understanding of exposure/disease pathways to more efficiently generate data to characterize potential health risks (often referred to as New Approach Methods (NAMs)). Toxicological research on chemical hazards is increasingly focused on mechanistic data and biological alterations that can lead to adverse health outcomes. As a method for incorporating this information into risk assessment, researchers have identified key characteristics of chemicals that have been linked to various hazard endpoints including cancer, reproductive toxicity, and endocrine disruption [74–78]. These key characteristics can be used to organize mechanistic data that can serve as early indicators of potential harm for chemicals and for early identification of hazard. While there is great opportunity to use these upstream markers of hazard, it is critical to recognize the important limitations of in vitro*, *in silico*,* and *in chemico* toxicology data and that these data cannot currently be used to make definitive determinations to rule out chemical health hazards, and should only be used to identify potentially hazardous chemicals and then address those hazards under the appropriate statute [79]. Expedited timelines to replace mammalian tests with high-throughput assays that are limited in their ability to provide critical information about health endpoints of concern, particularly for highly exposed and/or susceptible populations like workers, frontline community members, children, and pregnant women, are inconsistent with providing health protections for these populations. Thus, agencies such as EPA should ensure that a hazard classification is not weakened based on speculative or limited data, but rather use NAMs to provide “actionable evidence,” or a scientific basis for health protective actions. These uses could include: facilitating dose–response assessments to support regulatory standards; investigating the impact of complex chemical mixtures; identifying susceptible populations and quantifying differences in risk; investigating how non-chemical stressors interact with chemical exposures. This approach to the use NAMs is consistent with recommendations from other regulatory agencies such as California EPA [80].

Read-across techniques can also be used to make hazard inferences from chemicals that are similar based on toxicological profile, structural similarity etc. [81]. These approaches are further discussed in the companion paper on chemical classes by Maffini et al. in this issue. In addition, PBTK modeling is often used to calculate daily intakes or internal doses of environmental pollutants but requires some caution. While PBTK models are a useful tool for assessing distribution of chemicals in an organism and can inform future research needs, they can be overly complicated given the available data, potentially obscure underlying data inadequacies and may not accurately reflect the range of internal doses appropriately, and could be misused by those with financial conflicts of interest [82]. This is further discussed in the companion paper on exposure by Vandenberg et al. in this issue.

Several approaches would increase the availability of appropriate data. First, claims of confidential business information (CBI) have frequently been used by industry to shield potentially critical data from public view, resulting in lack of transparency which is foundational to meaningful involvement of the public in government decision making [35, 83]. CBI rationales should be evaluated carefully and reevaluated if necessary with a goal of increasing transparency and availability of data to ensure that claims are valid under the prevailing law, and further action should be taken to strengthen the laws to promote greater availability of data to the public if necessary. Additionally, EPA could use specific approaches to incentivize data generation including publishing provisional toxicity values for chemicals and applying multiple default adjustment factors as needed to account for lack of data. Approaches, such as using adjusted maximum tolerated doses and/or acute LD50 values, which are correlated with cancer potency, have been recommended by the National Academy of Sciences (NAS) based on empirical data [47, 84–88]. Timely policy remedies that incentivize data generation should be critical to both protecting the public’s health and to improved understanding and characterization of chemical risks. Examples of such adjustment factors are discussed in the companion paper on exposure by Vandenberg et al. and the companion paper on human variability by Varshavsky et al. in this issue.

Swift action should be taken to mitigate exposures to chemicals with hazardous properties at the first indication of potential harm to public health, even without a full risk assessment conducted or when evidence of harm is limited. In some instances, policy makers and public health officials have acted on early indication of harm in order to protect the public immediately without waiting for more robust evidence, which has resulted in decreased exposures and avoided adverse health effects [60, 67]. An example of such a model is the California Safer Consumer Products (SCP) regulations, which require the Department of Toxic Substances Control (DTSC) to only demonstrate a *potential* for exposures and significant or widespread adverse impacts before health-protective action is taken [89]. Under these regulations to identify priorities, DTSC does not need to conduct a formal risk assessment, nor a weight-of-evidence analysis; one reliable study alone that indicates such potential can suffice for DTSC to take action on a product or chemical of concern, requiring the responsible entities (often the product manufacturers) to either remove the chemical or product from the market or conduct an Alternatives Analysis to evaluate whether the chemical(s) in question can be replaced with a safer alternative. Depending on the results of the Alternatives Analysis, DTSC may then consider regulatory responses to protect public health and the environment, including disclosure of chemical use to consumers, limits on use, or sales bans.

#### 3. Populations at greater risk, including those that are more susceptible or more highly exposed, must be better identified and protected to account for their real-world risks

Exposure to environmental pollution and toxic chemicals, which disproportionately impacts the health of children, workers, low-wealth communities, and communities of color, is a preventable threat to public health recognized by academics and medical societies alike [17, 19, 23, 90]. To achieve health protection for all, we need to address health inequities – differences in health status or in the distribution of health resources between population groups, arising from the social conditions in which people are born, grow, live, work, and age. This includes identifying and prioritizing communities that are disproportionately impacted by environmental pollution due to structural inequities such as systemic racism [91, 92]. For example, the legacies in the U.S. of redlining and racial segregation contribute to current differential environmental contamination, such as in the case of lead exposures, which have been disproportionately higher in predominantly-Black communities [93]. Although many environmental laws aspire to protect health, the implementation of these laws and policies often fail to ensure equitable, socially just safeguards [94, 95].

This commitment to health equity was a central element of President Biden’s January 2021 *Modernizing Regulatory Review* memorandum, where he called on all federal government agencies to “take into account the distributional consequences of regulations…to ensure that regulatory initiatives appropriately benefit and do not inappropriately burden disadvantaged, vulnerable, or marginalized communities” [96]. In a separate executive order, President Biden urged agencies to develop “programs, policies, and activities to address the disproportionately high and adverse human health, environmental, climate-related and other cumulative impacts on disadvantaged communities” [97]. EPA has since developed an Equity Action Plan and Strategic Plan to implement these orders [35].

One area for EPA to advance this commitment is through implementation of amended TSCA as Congress intended: to improve the policy addressing harmful industrial chemicals that can exacerbate existing health disparities [34, 98]. Under amended TSCA, EPA has an obligation to protect potentially exposed or susceptible subpopulations, defined as *“*a group of individuals within the general population identified by the Agency who, due to either greater susceptibility or greater exposure, may be at greater risk than the general population of adverse health effects from exposure to a chemical substance or mixture” from any unreasonable risks as a result of chemical use [34]. To accomplish this EPA must account for factors that increase likelihood of chemical exposures or susceptibility to chemical exposures when identifying hazards, estimating exposures, and quantitatively estimating dose–response relationships. This has been done to some extent by other government agencies such as the California EPA, and is discussed further in the companion papers on human variability by Varshavsky et al. and exposure assessment by Vandenberg et al. in this issue.

Scientific evidence demonstrates that intrinsic factors (e.g., pre-existing disease, life stage, reproductive status, age, sex, genetics) and extrinsic factors (e.g., food insecurity, geography, socioeconomic status, racism/discrimination, cultural factors, workplace/occupation) impact susceptibility to or likelihood of environmental chemical exposures, leading to differential risks [99]. Populations that experience multiple social, physical, and chemical environmental stressors are at additional increased risk of disease. [13, 100–102]. However, the current definition EPA uses for these populations and its methodologies in TSCA risk evaluations do not fully consider all of these factors either separately or together (e.g., pregnant workers) and could result in EPA mischaracterizing populations and their susceptibilities, leading to underestimated or unidentified risks, and therefore underprotective risk management rules [39, 40]. EPA’s first ten existing chemicals risk evaluations under amended TSCA, completed in 2020, failed to adequately capture the full range of factors that influence susceptibility to chemical exposures [39, 40]. Additionally, when PESS such as workers were identified, EPA’s assessment incorrectly assumed the use of adequate personal protective equipment (PPE), which resulted in underestimation of worker risk and was characterized as scientifically unjustified by EPA’s peer review committee [103, 104].

EPA could and should incorporate a more robust definition of susceptible populations into its risk assessment policies and guidelines. We recommend a modified version (explicitly identifying racism and other extrinsic factors, and identifying that whole communities, rather than groups of individuals, can have increased exposure or susceptibility) of the definition found in EPA’s January 2017 proposed TSCA risk evaluation framework rule, which focused on identifying intrinsic and extrinsic factors of susceptibility [105, 106] (modifications to EPA’s proposed definition are in **bold**):

*Potentially exposed or susceptible subpopulation means a group of individuals* ***or communities*** *within the general population identified by the Agency who, due to greater susceptibility may be at greater risk than the general population of adverse health effects from exposure to a chemical substance or mixture, including but not limited to infants, children, pregnant* ***people****, workers, or the elderly.* ***Susceptibility can be due to either intrinsic (e.g., pre-existing disease****, life stage, reproductive status, age,* ***sex,*** *genetic traits)* ***or extrinsic*** *(e.g.,* ***food insecurity,*** *geography,* ***poverty, socioeconomic status, racism, discrimination, culture,*** *workplace)* ***factors when identifying this population*** [106, 107].

Explicitly naming factors that qualify groups as populations at greater risk (PESS under TSCA) is an important step to ensure their experiences are consistently addressed in hazard and risk assessments. EPA must actively identify and consult with community experts early and often during the risk assessment process, as they have intimate knowledge of chemical uses, exposures, and hazards in their communities due to unplanned or accidental releases, including from climate change. Additionally, these communities often generate data themselves, as their exposures may not be captured by databases that rely on estimates premised on ideal or normal operations. Both of these things make the information communities provide integral to scientific evaluations. Agencies can do this by publicly releasing accessible information about chemical risks to communities and collaborating with community representatives to better characterize exposures and risks. EPA must also utilize aggregate exposure and cumulative risk frameworks in all risk assessments [37]. The above factors can be applied beyond TSCA in other policy and regulations across federal agencies, including the federal guidance on regulatory analysis, Circular A-4 [107].

#### 4. Hazard and risk assessments should not assume existence of a “safe” or “no-risk” level of chemical exposure in the diverse general population

The default hazard and risk assessment approach used by government agencies such as EPA to evaluate health effects of chemical exposures for health outcomes other than cancer (e.g., reproductive, developmental, neurological and cardiovascular effects) is to assume there is a threshold, or a “safe” level of exposure, below which there is no (or negligible) risk of adverse health effects [47]. EPA operationalizes the threshold approach through the calculation of the oral reference dose (RfD) and the inhalation reference concentration (RfC), which are defined as levels of exposure “likely to be without an appreciable risk of deleterious effects” [46]. The term “appreciable risk” is not defined, and thus RfDs and RfCs are not associated with any specified quantitative level of risk. EPA uses the RfD and RfC as “bright lines” where any exposures below these values are effectively assumed to be without risk and thus are treated as being safe for human exposure. However, there are several fundamental scientific flaws with assuming a “safe” level of exposure to chemicals of concern and this approach therefore often leads to public health policies that fail to sufficiently protect individuals, groups, or communities, especially PESS. Chemical exposures at or above the level of the RfD or RfC are assumed to pose some concern, but the RfD/RfC approach does not provide quantitative information about the level of risk for non-cancer health effects at varying exposure levels above or below the threshold and can obscure the true health impacts of chemical exposures. Additionally, lack of risk quantification means that these health effects are not included in quantitative benefits assessments, a significant barrier to full consideration of noncancer health outcomes in chemicals policy decisions [47, 51].

The threshold approach rests on the assumption that physiological defense systems and repair mechanisms can overcome any adverse effects of low-dose exposure and that everyone in the population has a similar vulnerability to exposure or capacity to respond [47, 108]. However, as discussed above in Principle 3, the diverse human population is comprised of individuals who have varying health susceptibilities from intrinsic (e.g., pre-existing disease, life stage, reproductive status, age, sex, genetics) and extrinsic (e.g., food insecurity, geography, poverty, socioeconomic status, racism, discrimination, access to care, culture, workplace) factors and are simultaneously exposed to *multiple* industrial chemicals through *multiple* pathways (e.g., air, food, water, products) in *multiple* locations (e.g., work, home, school) [36]. These susceptibilities can impair the ability of physiological defenses to respond to low-dose exposures – with the degree of impairment varying substantially among individuals. A chemical’s non-carcinogenic effects may be associated with a threshold for any given individual, but the level of this individual threshold is likely to vary across a diverse population in which there is a high likelihood for interactions with common background disease process and chemical co-exposures [47, 109]. Thus, some risk of an adverse health effect can be expected at low and observable levels of exposure across the diverse population. Further, as observed by the NAS*,* there are a variety of non-cancer risks with no evidence of a threshold when studied across diverse human populations, with prime examples being lead and particulate matter [47].

While results of experimental laboratory animal studies may appear to suggest the existence of a level at which there is no adverse effect, this interpretation is often invalid due to limitations inherent in the study design, such as small sample size, lack of genetic diversity, lack of diversity in underlying health status, insensitivity of outcomes assessed, lack of variability in background exposures to other chemicals, and inadequate statistical models of the data [43, 110, 111]. EPA has determined that doses identified in toxicology studies as “no observed adverse effect levels” (NOAELs) typically have a response rate of “about 5–20% on average, not 0%” and that, due to the limitations of laboratory studies, the NOAEL “does not represent a biological threshold and cannot establish that lower exposure levels are necessarily without risk” [43]. These limitations mean that laboratory studies frequently fail to capture the variability in real-world intrinsic and extrinsic factors that influence the range of human responses to a chemical exposure in a diverse exposed human population, and thus are limited in capturing risks to those that are more susceptible. These limitations are a barrier to identifying the occurrence of adverse outcomes (or their biological precursors) in humans at environmentally-relevant exposure levels.

The variability in characteristics affecting an individual’s response to chemical exposure results in a wide range of individual thresholds and an expectation of dose–response relationships in the population that extend to low, commonly experienced doses, with probability of risk at doses below the traditional RfD and RfC. It therefore cannot be presumed that low levels of exposure are risk-free. It should instead be assumed that low levels of exposure are associated with some level of risk, unless there are sufficient data to indicate a threshold level below which there is no risk for the entire exposed population [47]. In place of the traditional RfD and RfC, we recommend applying methods proposed by authoritative bodies such as the NAS and WHO to quantify risks of non-cancer effects and derive risk-specific doses, as demonstrated in published case studies [47, 50, 112–114]. These methods are further discussed and demonstrated in the companion paper by Nielsen et al. in this issue.

#### 5. Hazard and risk assessments must evaluate and account for financial conflicts of interest in the body of evidence

It is well established that chemical industry sponsors and researchers financially supported in whole or part by the chemical industry gain from asserting that industrial chemicals are safe and sowing doubt about data to the contrary [58, 115]. These findings can be used to prevent, delay, alter, or minimize regulation, and market these chemicals to increase their production and sale and such tactics have been used in various hazardous agents including but not limited to asbestos, lead, and tobacco [59, 115, 116]. In contrast to independent scientists and public health practitioners who do not generate economic profit, the incentive to profit from a research outcome may lead industry and industry-sponsored scientists to alter research practices in ways that can bias findings regarding the harms of these chemicals, including how they frame the research questions, design and conduct a study, code events, analyze the study data, report the results, and characterize conclusions [54, 117–120]. For example, EPA’s Office of Pesticide Programs (OPP) noted important differences in reported outcomes based on study sponsorship in a 2019 evaluation of data linking exposure to the herbicide paraquat with Parkinson’s disease risk [121]. EPA highlighted that industry-sponsored studies "mostly present null results using an exposure design similar to studies in the literature that report significant decline in dopaminergic neuron counts." Scientists from the U.S. National Toxicology Program (NTP) reviewed the same data set and found that established adverse outcomes were unlikely to be demonstrated in the industry-sponsored studies as the duration of the study was “too short and dosing too infrequent” to reliably cause observable adverse effects [121].

EPA offices and authoritative bodies in Europe that conduct hazard identification and risk assessment use a variety of methods to assess potential risks of bias in primary research studies, but these do not assess one potential source of bias—financial conflicts of interest (COI) [27, 122, 123].

Even when controlling for the methodological risk of bias or internal validity, instances when studies are determined to have similar biases based on evaluation of their published methods (e.g., how they conducted exposure and outcome assessment), studies with industry sponsorship or authors with a financial COI are more likely to report findings that favor the sponsor’s product than those without [58, 124–128]. This phenomenon occurs across several research areas including tobacco, pharmaceutical, nutrition, and chemical. For example, in a study examining whether industry research sponsorship is associated with bias in the methods, results, or conclusions of animal studies examining the effect of exposure to atrazine on reproductive or developmental outcomes, no differences were found in methodological risks of bias (internal validity) in industry versus non-industry sponsored studies. Nonetheless, industry sponsored studies were less likely to conclude that atrazine was harmful compared to non-industry studies [127]. Disregarding sponsorship and solely examining the methods therefore is not sufficient to capture the potential for bias in a study.

This empirically demonstrated bias highlights why reliance on industry-supported GLP (Good Laboratory Practice) compliant toxicology studies, used almost exclusively by pesticide regulators, must also be addressed [32]. Although GLP standards have improved record keeping of commercial laboratories, it has been established they are not a valid measure of internal validity, as they specify nothing about the quality of the research design, the skills of the technicians, the sensitivity of the assays, or whether the methods employed are current or out-of-date [129]. Therefore GLP standards should not be the sole basis for regulatory decision making, and their methodological biases should be accounted for in any evidence evaluation [130]. A critical example of this is the oncogenicity of the herbicide glyphosate, a chemical that EPA determined was “not likely to be carcinogenic to humans” in 2015, in contrast with the International Agency for Research on Cancer (IARC) Group 2A classification of “probably carcinogenic to humans” that same year [32]. In reaching these divergent conclusions, EPA relied almost exclusively on industry-conducted, GLP-compliant unpublished regulatory studies, of which, 99% were negative, while IARC relied mostly on peer-reviewed studies, 70% of which were positive; EPA's analysis was found to be fatally flawed by the US 9th Circuit Court of Appeals [131, 132]. The NASEM 2021 *Review of the EPA's ORD Staff Handbook for Developing IRIS Assessments* highlighted a need to quantify the influence of funding source on GLP studies conducted by contract research organizations (CROs) [133].

The discrepancy in the outcomes and interpretations of industry-sponsored studies compared with non-industry-sponsored studies is of concern as rigorous hazard and risk assessments depend on an evidence base that is as free of bias as possible to ensure chemical harms are not underestimated. Thus, hazard and risk assessment conclusions should account for the potential influence of industry sponsorship and author COI. This can be accomplished in systematic reviews by including a separate domain for funding source and author COI when evaluating individual study risk of bias, which was recommended by the NAS in its 2014 review of the EPA Integrated Risk Information System (IRIS) program’s systematic review methodology. The NAS concluded that “Funding sources should be considered in the risk-of-bias assessment conducted for systematic reviews that are part of an IRIS assessment” and expanded on this point in its 2021 review of the IRIS handbook, saying that EPA “should describe how to detect and assess the effect of funding bias on the confidence of study ratings from evidence evaluation or effect estimates from synthesis” [133, 134].

US government agencies, such as EPA, and leading European authoritative bodies should therefore amend their current risk of bias assessment tools to include industry sponsorship and author COI as a separate risk of bias domain [27, 122, 123]. Importantly, including funding as a risk of bias domain does not require automatically excluding or discounting industry sponsored studies from EPA’s hazard and risk assessment; it means documenting funding as one of many domains of potential bias and its impact on the overall quality of the body of evidence as part of the hazard evaluation process.

## Conclusion and next steps

This consensus statement and the subsequent papers create an evidence-based foundation for improved approaches to evaluating and using the science related to toxic chemicals and their potential health impacts. We have seen the growth of chemical production, manufacturing and industrialization continue with few constraints and we have repeated examples of how insufficient data and evaluation of science have enabled widespread exposures and demonstrated adverse health effects often disproportionately experienced by children, workers, low-wealth communities, and communities of color. More robust science generation and evaluation coupled with shifts in regulatory and economic policies to strengthen public health protective actions are part of addressing and preventing these harms. Lead in gasoline and paint, asbestos in insulation, and radiation exposure are three among numerous examples that illustrate the public health consequences of delays or failure to act on the best available scientific data when making policy and regulatory decisions [60, 67]. The analyses in these manuscripts build on recommendations already agreed upon by authoritative bodies, used by some US states and other nations, and represent decades of subject-matter expertise. They also build on comprehensive precautionary strategies already being pursued in Europe, such as the EU’s chemicals strategy for sustainability towards a toxic-free environment [135]. Our recommendations are only one element of an overall improved and integrated approach to managing chemicals and pollution and their swift incorporation is critical to make necessary improvements within the current existing chemical evaluation and management structure in the US [136]. Strengthened hazard and risk assessments – in combination with consideration of other factors such as values and preferences, costs and benefits, essentiality, availability of safer alternatives, and uncertainties – will contribute to policy solutions to improve population health and eliminate health disparities, many of which are the product of systemic racism. Ultimately, these steps forward will better inform environmental health decision-making and lead to improved, more equitable, public health protection.

## Data Availability

Data sharing is not applicable to this article as no datasets were generated or analyzed during the current study.

## Abbreviations

CAA: Clean Air Act
CAS: Chemical Abstracts Service
CBI: Confidential Business Information
COI: Conflicts of Interest
CPSC: Consumer Product Safety Commission
CRO: Contract Research Organization
DTSC: Department of Toxic Substances Control
ECHA: European Chemials Agency
EFSA: European Food Safety Authority
EPA: Environmental Protection Agency
EU: European Union
FDA: Food and Drug Administration
FIFRA: Federal Insecticide, Fungicide, and Rodenticide Act
GLP: Good Laboratory Practice
IARC: International Agency for Research on Cancer
IRIS: Integrated Risk Information System
NAMs: New Approach Methods
NAS: National Academy of Sciences
NASEM: National Academies of Sciences, Engineering, and Medicine
NOAELs: No Observed Adverse Effect Levels
NTP: National Toxicology Program
OPP: Office of Pesticide Programs
OSHA: Occupational Safety and Health Administration
PCBs: Polychlorinated Biphenyls
PESS: Potentially Exposed or Susceptible Subpopulations
PPE: Personal Protective Equipment
REACH: Registration, Evaluation, Authorisation and Restriction of Chemicals
RfC: Reference Concentration
RfD: Reference Dose
SCP: Safer Consumer Products
SDWA: Safe Drinking Water Act
TSCA: Toxic Substances Control Act
US: United States
WHO: World Health Organization
